# The Structure of the Lipid A from the Halophilic Bacterium *Spiribacter salinus* M19-40^T^

**DOI:** 10.3390/md16040124

**Published:** 2018-04-11

**Authors:** Clara Barrau, Flaviana Di Lorenzo, Rodolfo Javier Menes, Rosa Lanzetta, Antonio Molinaro, Alba Silipo

**Affiliations:** 1 Department of Chemical Sciences, University of Naples Federico II, 80126 Naples, Italy; clarabarrau@gmail.com (C.B.); lanzetta@unina.it (R.L.); molinaro@unina.it (A.M.); 2 Cátedra de Microbiología, Facultad de Química y Unidad Asociada de Facultad de Ciencias, Universidad de la República, 11800 Montevideo, Uruguay; jmenes@fq.edu.uy

**Keywords:** *Spiribacter salinus*, halophiles, *Ectothiorhodospiraceae*, lipopolysaccharide (LPS), lipid A, matrix-assisted laser desorption ionization (MALDI) mass spectrometry, 3-oxotetradecaonic acid

## Abstract

The study of the adaptation mechanisms that allow microorganisms to live and proliferate in an extreme habitat is a growing research field. Directly exposed to the external environment, lipopolysaccharides (LPS) from Gram-negative bacteria are of great appeal as they can present particular structural features that may aid the understanding of the adaptation processes. Moreover, through being involved in modulating the mammalian immune system response in a structure-dependent fashion, the elucidation of the LPS structure can also be seen as a fundamental step from a biomedical point of view. In this paper, the lipid A structure of the LPS from *Spiribacter salinus* M19-40^T^, a halophilic gamma-proteobacteria, was characterized through chemical analyses and matrix-assisted laser desorption ionization (MALDI) mass spectrometry. This revealed a mixture of mono- and bisphosphorylated penta- to tri-acylated species with the uncommon 2 + 3 symmetry and bearing an unusual 3-oxotetradecaonic acid.

## 1. Introduction

Extremophiles are organisms that can live in environments that are considered to be destructive for many other living forms. Bacteria that possess these properties represent an appealing source of metabolites with a wide range of biotechnological applications [1,2]. Moreover, the study of the adaptation phenomena of such microorganisms would help researchers to appreciate their survival mechanisms, which, in turn, would help to understand the process by which their structural elements (i.e., proteins, genes, and glycolipids) could be altered and employed for therapeutic implications [3].

In this context, halophiles—namely organisms that are able to live and proliferate in high saline habitats—represent a promising reservoir for the isolation of new biologically active compounds. Indeed, hypersaline environments are enriched with bacteria that provide a copious source of natural substances [4,5], including antibiotics, antitoxins, antitumoral compounds, and enzymes [6].

*Spiribacter salinus* (*S. salinus*) M19-40^T^ is a halophilic, Gram-negative gamma-proteobacterium that has been recently identified as a new genus, based on a metagenomics analysis with close phylogenetic similarities with species of the genera *Alkalilimnicola*, *Alkalispirillum*, and *Arhodomonas* within the family *Ectothiorhodospiraceae*. It was first isolated from an intermediate-salinity pond of a marine saltern—located in Isla Cristina, Huelva, Southwest Spain [7].

As a Gram-negative bacterium, *S. salinus* M19-40^T^ possesses lipopolysaccharides (LPSs) as the major component of its outer membrane (OM). LPSs are amphiphilic macromolecules that are composed of three structural domains: a polysaccharide termed O-antigen, a core oligosaccharide, and a glycolipid portion—the lipid A—embedded in the OM. An LPS showing all three domains is defined as a smooth-type LPS (S-LPS), whereas when the O-antigen is absent, the terminology employed is a rough-type LPS (R-LPS or LOS) [8,9,10]. Structurally, the lipid A is typically composed of a β-(1→6)-d-glucosamine disaccharide backbone, which is phosphorylated—by primary and secondary acyl chains—at positions 1 and 4′ and acylated at positions 2 and 3 of each glucosamine [11,12]. LPSs are known to interact with mammalian innate immune systems, as their lipid A moiety is specifically recognized by the Toll-Like Receptor 4/myeloid differentiation protein-2 (TLR4/MD-2) receptorial complex, which triggers the production, inflammation, and adaptive immune response of pro-inflammatory cytokines [13]. The immunopotency of an LPS is strictly structure-related. In particular, structural variations of the lipid A, such as diversity in the acylation pattern, in the length and chemical structure of fatty acids, and in the modification of phosphate groups, are associated with different immunological activity. Indeed, depending on the structure of the lipid A part, an LPS can act either as a TLR4/MD-2 agonist, activating the inflammatory response, or as an antagonist, preventing the binding of toxic LPSs. Consequently, an LPS can limit the dangerous effects that are caused by such an interaction. The activation of TLR4 by non-toxic LPS variants is considered as an ingenious approach towards potent and selective immunomodulators that are to be used as immune-therapeutics [11,12,13,14,15]. On the other hand, natural LPS variants, or synthetic derivatives that are able to inhibit the activation of TLR4 by competing with toxic LPS or other agonists for TLR4/MD-2, are attractive candidates for drugs targeting pathogens that are caused by excessive TLR4 activation upon stimulation by bacterial LPS (sepsis and septic shock) [11,12,13,14,15].

Given these premises—and considering that the LPS structure is strongly influenced by the physiological conditions of the surrounding environment—it is expected that halophilic bacteria, such as *S. salinus* M19-40^T^, modify their LPS architecture to colonize the hostile habitat, thus reinforcing the membrane and assuring physical protection. Therefore, LPSs that express uncommon structural features are expected for halophiles. In this context, LPSs with unusual structural characteristics are considered as potential antagonists of the TLR4/MD-2 complex [16].

In this scenario, the structural characterization of LPSs—particularly that of the lipid A part—from halophiles is the mandatory starting point for understanding the environment adaptation phenomena. However, this also opens to the assessment of the structure–function relationship in a perspective of the conception of new generation drugs.

This study reports the characterization of *S. salinus* M19-40^T^ lipid A structure. This is achieved by merging information that was attained from the compositional analyses performed on pure LPS with information from a matrix-assisted laser desorption ionization (MALDI) mass spectrometry (MS) and MS^2^ investigation executed on the isolated lipid A fraction.

## 2. Results

### 2.1. Isolation and Compositional Analysis of the Lipid A from S. salinus M19-40^T^ LPS

LPS material was extracted from dried bacterial cells using the hot phenol/water procedure [17]. The LPS material was found only in the water phase and underwent an enzymatic treatment in order to remove cell contaminants, such as nucleic acids and proteins. A sodium deoxycholate-polyacrylamide gel electrophoresis (DOC-PAGE), followed by silver nitrate staining [18], was performed, revealing the rough nature (R-LPS) of the extracted material. An additional purification step was performed by ultracentrifugation, followed by size-exclusion chromatography.

The compositional analysis executed on R-LPS revealed that *S. salinus* M19-40^T^ lipid A was mainly composed of (*R*)-3-hydroxydecanoic acid (10:0 (3-OH)), (*R*)-3-hydroxytetradecanonic acid (14:0 (3-OH)), 3-oxo-tetradecanonic acid (14:0 (3-oxo)), and dodecanoic acid (12:0). Decanoic (10:0), undecanoic (11:0), tridecanoic (13:0), tetradecanoic (14:0), and tetradecenoic (14:1) acids were also detected as minor species.

**Figure 1:**
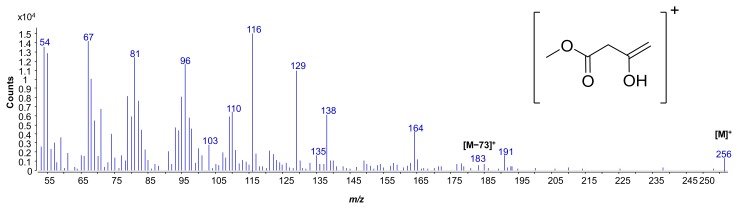
Mass spectrum of the methyl 3-oxotetradecanoate. In the inset, the species at *m*/*z* 116 originating from the beta cleavage to the oxo group, and containing the carboxyl moiety, is sketched.

The presence of the 14:0 (3-oxo) fatty acid was confirmed by the analysis of the fragmentation pattern of its methyl ester derivative (Figure 1) as follows: (i) The presence of the molecular ion peak at *m*/*z* 256 and (ii) the presence of the base peak at *m*/*z* 116, which clearly proved the beta cleavage to the oxo group with the rearrangement of one hydrogen atom [19]. Moreover, (iii) the minor peak at *m*/*z* 183 [M − 73]^+^, which was indicative of the alpha cleavage to the oxo group, was also identified [20]. The occurrence of the 14:0 (3-oxo) fatty acid could not be shown using the typical procedure to define the total fatty acid content [21], most likely because of decarboxylation [20]. On the contrary, the liberation of the fatty acids by methanolysis with methanol/hydrochloric acid revealed the occurrence of 14:0 (3-oxo).

### 2.2. MALDI MS and MS^2^ Analysis on the Isolated Lipid A from S. salinus M19-40^T^ LPS

An aliquot of pure R-LPS underwent a mild acid hydrolysis with an acetate buffer in order to selectively cleave the acid labile linkage between the core oligosaccharide and the lipid A. The isolated lipid A fraction then underwent a detailed MALDI MS and MS^2^ investigation.

**Figure 2:**
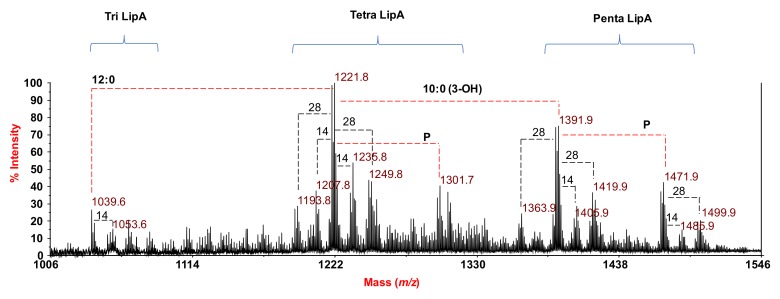
Reflectron matrix-assisted laser desorption ionization (MALDI) mass spectrum, recorded in negative polarity, of lipid A from *S. salinus* M19-40^T^ lipopolysaccharides (LPS) that were obtained after acetate buffer treatment. Only deprotonated ions [M − H]^−^ are formed in these conditions. The lipid A species are indicated.

The reflectron MALDI mass spectrum, which was recorded in negative polarity, is presented in Figure 2. The spectrum clearly indicated—in the range *m*/*z* 1039.6–1499.9—a complex pattern of peaks relative to deprotonated [M − H]^−^ lipid A species, which differed in both the nature and number of fatty acids and in phosphate content. Three main clusters of peaks were detected and matched with penta-, tetra-, and tri-acylated lipid A species, whose heterogeneous nature was also clearly visible because of the presence of mass differences of 14 (–CH_2_– unit) and/or 28 amu (–CH_2_CH_2_– unit), which are diagnostic for lipid A species that differ in the length of their acyl chains, in accordance with compositional analysis data.

In detail, the cluster of peaks at approximately *m*/*z* 1391.9 and *m*/*z* 1471.9 were assignable to mono- and bisphosphorylated penta-acylated lipid A species (Figure 2) carrying two 10:0 (3-OH), one C14:0 (3-OH), one C14:0 (3-oxo), and one C12:0. In the mass range *m*/*z* 1193.8-1301.7, tetra-acylated lipid A species were identified: The peak at *m*/*z* 1301.7 was matched with bisphosphorylated tetra-acylated lipid A species carrying one 10:0 (3-OH), one 14:0 (3-OH), one 14:0 (3-oxo), and one 12:0. The corresponding monophosphorylated form was assignable to the peak at *m*/*z* 1221.8. In the same mass range, monophosphorylated lipid A species that differed in the length of the acyl chains were clearly visible because of the occurrence of mass differences of 14 and 28 amu, as stated above. Finally, monophosphorylated tri-acylated lipid A species that were lacking in both 10:0 (3-OH) and 12:0, with respect to the penta-acylated lipid A species at *m*/*z* 1391.9, were also visible at *m*/*z* 1039.6 (Figure 2).

**Figure 3:**
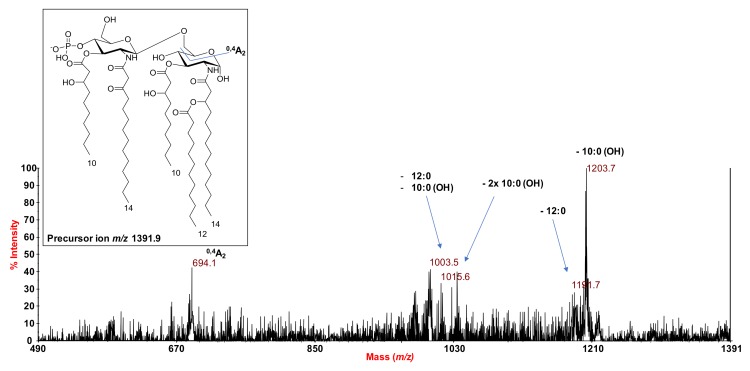
MALDI mass spectrometry (MS)/MS^2^ spectrum of the monophosphorylated penta-acylated lipid A species at *m*/*z* 1391.9 from *S. salinus* M19-40^T^ LPS. The fragments’ assignment is reported. The proposed structure for the penta-acylated lipid A species is reported in the inset.

To define the detailed structure of *S. salinus* M19-40^T^ lipid A, unveiling the location of the acyl moieties with respect to the glucosamine disaccharide backbone, an MS^2^ analysis on several peaks was performed. In detail, the negative-ion MS^2^ spectrum of the precursor ion at *m*/*z* 1391.9 (Figure 3), corresponding to a monophosphorylated penta-acylated lipid A species, showed an intense peak at *m*/*z* 1203.7. This was attributed to an ion that was derived from the loss of a 10:0 (3-OH) fatty acid. A less intense peak was also observed at *m*/*z* 1191.7 and was assigned to a fragment that originated from the loss of one 12:0 unit. Further minor peaks at *m*/*z* 1003.5 and *m*/*z* 1015.6 were identified and assigned as follows: (i) the ion at *m*/*z* 1003.5 was matched with a fragment that was caused by the sequential loss of both one 10:0 (3-OH) and one 12:0 unit, whereas (ii) the ion at *m*/*z* 1015.6 corresponded to a lipid A piece lacking two 10:0 (3-OH) moieties. The observation of both of these fragmentations was fundamental for the structural characterization. Indeed, the presence of a fragment that originated from a sequential loss of 10:0 (3-OH) and 12:0 from the precursor ion—in addition to the absence of fragmentations that referred to the loss of a whole unit of a hydroxylated fatty acid bearing a secondary acyl substituent—concurred to indicate that the secondary 12:0 was bound to an N-linked primary fatty acid. Moreover, the occurrence of the ion at *m*/*z* 694.1, originating from the sugar ring fragmentation (^0,4^A_2_) [22], was crucial to establish the nature of the primary fatty acids that decorated the non-reducing glucosamine unit (that is, one 10:0 (3-OH) and one 14:0 (3-oxo)), and thus to locate the secondary acyl chain 12:0 only at the reducing glucosamine unit.

**Figure 4:**
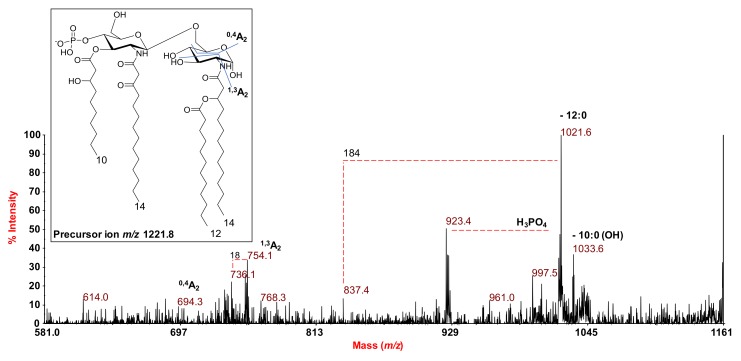
MALDI MS^2^ spectrum of the monophosphorylated tetra-acylated lipid A species at *m*/*z* 1221.8 from *S. salinus* M19-40^T^ LPS. The fragments’ assignments are reported. The proposed structure for the tetra-acylated lipid A species is reported in the inset.

The MS^2^ analysis of the precursor ion at *m*/*z* 1221.8 (Figure 4), corresponding to a tetra-acylated and monophosphorylated species, indicated an intense peak at *m*/*z* 1021.6, which was attributed to an ion that was derived from the loss of the 12:0 moiety. A subsequent rearrangement of the ion at *m*/*z* 1021.6, promoted by the free 3-OH group on the 14:0, gave a loss of 184 mass units (C_12_H_24_O), resulting in the ion at *m*/*z* 837.4 (Figure 4) [23]. This fragmentation was helpful to establish that the primary 14:0 (3-OH) was sitting at position 2 of the reducing glucosamine. Moreover, the observation of such fragmentation, which occurred only when the secondary 12:0 unit was absent, suggested that the latter could be linked to the 14:0 (3-OH) moiety. A further intense peak, at *m*/*z* 923.4, was assigned to a fragment devoid of both the 12:0 unit and the phosphate group. A minor peak was also observed at *m*/*z* 1033.6, matching with a fragment that originated from the loss of a 10:0 (3-OH) acyl moiety (Figure 4). Two ions, derived from sugar ring fragmentations ^0,4^A_2_ (*m*/*z* 694.3) and ^1,3^A_2_ (*m*/*z* 754.1), were also detected. In particular, cross-ring fragmentation ^1,3^A_2_ (*m*/*z* 754.1), which only took place when the hydroxyl group at position 3 of the reducing glucosamine was not substituted, further confirmed that the missing 10:0 (3-OH) acyl chain was placed in that position, and thus that 14:0 (3-OH) and 12:0 were located at position 2 of the same glucosamine as the primary and secondary fatty acids, respectively.

The MS^2^ analysis of the precursor ion at *m*/*z* 1301.7 (Figure S1), relative to a bisphosphorylated tetra-acylated lipid A species, confirmed the structural characterization. This was because the occurrence of both the Y_1_ ion (*m*/*z* 666.3)—which originated from the cleavage of the glycosydic linkage of the glucosamine backbone—and the ring fragmentation-derived ion ^1,3^A_2_ (*m*/*z* 834.6) clearly indicated that the reducing glucosamine was decorated by 14:0 (3-OH) and 12:0, whereas the non-reducing glucosamine was acylated by 10:0 (3-OH) and 14:0 (3-oxo). Once again, the loss of 184 mass units was detected only from a fragment that was lacking in the secondary 12:0 unit.

**Figure 5:**
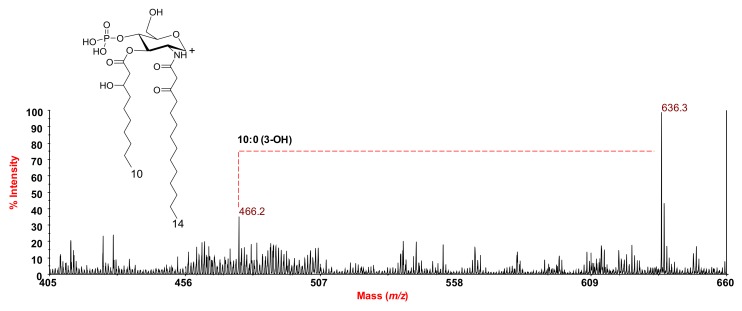
Section of the reflectron MALDI mass spectrum, recorded in positive polarity, of lipid A from *S. salinus* M19-40^T^ LPS obtained after acetate buffer treatment. The oxonium ion structure is also sketched.

The positive-ion MALDI MS spectrum confirmed the presence of the molecular mass of the lipid A species with Na^+^ counterions (Figure S2). A very diagnostic peak for structural elucidation was detected at *m*/*z* 636.3 (Figure 5) and attributed to the oxonium ion. This important fragment ion arose from the cleavage of the glycosydic linkage and was indicative of the substitution on the non-reducing glucosamine unit, which confirmed that it bore one 14:0 (3-oxo) and one 10:0 (3-OH). Finally, a further peak at *m*/*z* 466.2 was identified and assigned to the oxonium ion that was lacking the 10:0 (3-OH) unit (Figure 5).

Finally, in order to unambiguously define the composition and position of the secondary fatty acids, an aliquot of lipid A underwent an ammonium hydroxide hydrolysis. The negative-ion MALDI mass spectrum (Figure S3) of the ammonium-treated lipid A displayed the following peaks that were important for the structural assessment, particularly in the mass range *m*/*z* 869.1–1051.2: (i) the ion at *m*/*z* 1051.2, which was explained as a monophosphorylated tri-acyl residue that was composed of both the primary N-linked 14:0 (3-OH) and 14:0 (3-oxo), with the former carrying the secondary 12:0 unit, and (ii) the ion at *m*/*z* 869.1, which was attributed to the monophosphorylated di-acyl residue that was composed only of the N-linked primary acyl moieties 14:0 (3-OH) and 14:0 (3-oxo). The occurrence of both of these peaks further corroborated the nature of the amide-bound acyl moieties and the occurrence of 12:0 as a secondary fatty acid in an acyloxyacyl amide moiety.

**Figure 6:**
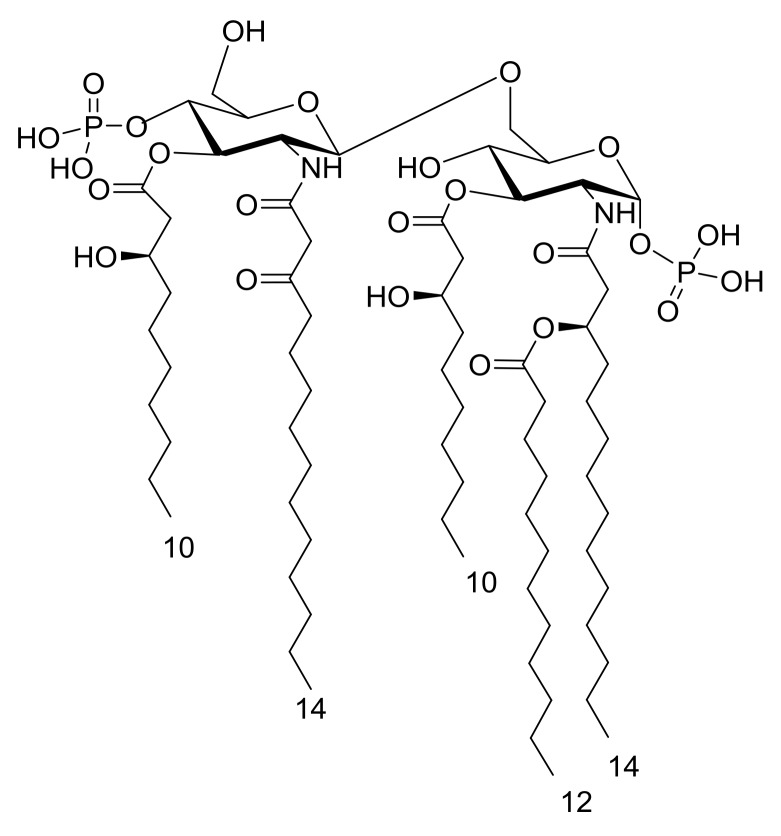
Proposed structure of *S. salinus* M19-40^T^ bisphosphorylated penta-acylated lipid species. The anomeric configuration of both of the glucosamine residues is putative, but in agreement with all of the lipid A species determined so far.

Thus, through a combination of analytical organic chemistry, and the merging of MS and MS^2^ data of the mild acid hydrolysis and the ammonium hydroxide-treated products with those from the compositional analysis, it was possible to define the *S. salinus* M19-40^T^ LPS as expressing a complex blend of the lipid A species—of which the component with the highest acylation degree is sketched in Figure 6.

## 3. Discussion

The adaptation mechanisms of marine bacteria to the different chemical, physical, and biological conditions that are encountered in marine environments have led to the evolution of highly diverse microbes. The adaptation phenomena typically involve the chemical modification of the bacterial membrane system in order to reinforce the overall cell envelope to resist the mutable conditions of the saline habitats. The structure of the halophilic microbes LPS often presents unusual chemical characteristics, which help the bacterium in maintaining membrane integrity. In particular, the nature and distribution of the fatty acids composing the lipid A moiety may change according to the external habitat.

In this paper, the characterization of the structure of the lipid A moiety of the R-LPS that was isolated from the slightly halophilic bacterium *S. salinus* M19-40^T^ is reported. The structural elucidation was performed, through MALDI MS and MS^2^ analysis, on the isolated lipid A. In parallel, a detailed fatty acid compositional analysis was executed on the LPS, providing the chemical constituents of the lipid A, which are essential to interpret the information derived from the MALDI MS investigation. *S. salinus* M19-40^T^ appeared to possess an R-LPS with a heterogenous lipid A, characterized by a mixture of tri-, tetra-, and penta-acylated species, decorated by one or two phosphate groups. In general, the 14:0 (3-OH) fatty acid was always found to be acyloxyamide linked to the reducing glucosamine; whereas 14:0 (3-oxo) was always found to be acyloxyamide decorating the non-reducing glucosamine; position 3 and 3′ were substituted with 10:0 (3-OH); and finally, the 12:0 unit was identified as a secondary substituent only of the reducing glucosamine. As indicated in Figure 6, the main penta-acylated lipid A species exhibits a 2 + 3 symmetry. Such an uncommon fatty acid distribution is interesting as most of the penta-acylated lipid A species—whose structure has been characterized to date—show a 3 + 2 asymmetry [11]. The unusual distribution of acyl substituents over the backbone resembles that of the lipid A of two psychrophilic and moderately halophilic bacteria, namely *Marinomonas vaga* [24] and *Marinomonas communis* [25], even though the chain length of the fatty acids is different. Moreover, *M. vaga* and *M. communis* possess only monophosphorylated species, and their 2 + 3 asymmetry is given by both the occurrence of a secondary acyl substitution on the acyloxyamide of the non-reducing glucosamine and by the absence of the primary ester-bound fatty acid decorating the same glucosamine unit.

Even more interestingly, *S. salinus* M19-40^T^ lipid A presented the rare amide-linked 14:0 (3-oxo), whose presence is consistent with the close phylogenetic similarities with species within the family *Ectothiorhodospiraceae*. Indeed, previous studies revealed that the LPS isolated from the extremely halophilic *Ectothiorhodospira halophila* also expresses the amide-bound 14:0 (3-oxo) fatty acids. Moreover, similarities with other *Ectothiorhodospira* species in lipid A fatty acid composition have been highlighted [26].

Given that, depending on the nature and distribution of the acyl chains, the lipid A may induce a TLR4/MD-2 agonistic or antagonistic activity after interaction with the host immune cells’ receptors, it is worth emphasizing that the presence of the 3-oxo fatty acids has also been shown for lipid As from *Rhodobacter sphaeroides* and *R. capsulatus*, two phototrophic bacteria that are of enormous importance in the LPS research field. Indeed, these bacteria exhibited a penta-acylated lipid A with strong TLR4/MD-2 antagonistic activity against toxic LPS and were used as an inspiration model for synthesis of the analogue Eritoran: a well-tolerated, synthetic lipid A mimetic, acting as a TLR4/MD-2 antagonist, which reached phase III clinical trials as an antisepsis agent [11].

Therefore, given the peculiar elucidated structure, the immunological activity of the lipid A from *S. salinus* M19-40^T^ LPS is also worth being investigated, since it can potentially provide interesting activities in the perspective of the development of analogues with an immunomodulatory action on the immune system.

## 4. Materials and Methods

### 4.1. Bacterial Growth 

The type strain *S. salinus* M19-40^T^ (LMG 27464^T^) was obtained from Belgian Coordinated Collections of Microorganisms (BCCM (Gent, Belgium)). The bacterial strain was grown in modified growth medium (MGM18) broth [27] at 30 °C in an orbital shaker (200 rpm) and biomass was harvested after 15 days by centrifugation (10,000× *g*).

### 4.2. Extraction and Purification of S. salinus M19-40^T^ LPS

The extraction of LPS was performed on lyophilized bacterial cells, following the hot phenol/water procedure [17]. The prolonged dialyses (Spectra/Por^®^ cut-off 12–14 kD, Ravensburg, Germany) against distilled water were then performed. The enzymatic treatment was executed to remove cell contaminants by using DNase (DN25-Sigma Aldrich^®^, St. Louis, MO, USA), RNase (R5503-Sigma Aldrich^®^) (37 °C, 5 h), and protease (P4630-Sigma Aldrich^®^) (56 °C, 16 h). The digested material was then extensively dialyzed (Spectra/Por^®^ cut-off 12–14 kDa) against distilled water. The nature of the partially purified material was determined by DOC-PAGE (Sigma Aldrich®, St. Louis, MO, USA), which was followed by gel staining with silver nitrate [18]. After lyophilization, the product was further purified by ultracentrifugation (Beckman Coulter’s ultracentrifuge (Brea, CA, USA), 4 °C, 45,000 rpm, 48 h) and then by size-exclusion chromatography on a Sephacryl High Resolution S-200 (GE Healthcare, Little Chalfont, UK) column. An aliquot of the pellet was then treated by mild acidic hydrolysis with an acetate buffer (pH 4.4, 2 h, 100 °C) in order to isolate the lipid A. A mixture of chloroform and methanol was added to the hydrolysis product to reach a chloroform/methanol/hydrolysate 2:2:1.8 (*v*/*v*/*v*) ratio. The mixture was then shaken and centrifuged. The chloroform phase, containing the lipid A, was collected and washed with the water phase of a freshly prepared Bligh/Dyer mixture (chloroform/methanol/water, 2:2:1.8) [28]. The organic phases were pooled, dried, and analyzed by MALDI MS (SCIEX, Concord, ON, Canada).

### 4.3. Chemical Analysis

The total fatty acid content was defined for pure LPS by treating it with 4 M HCl (100 °C, 4 h), followed by a treatment with 5 M NaOH (100 °C, 30 min). After the adjustment of the pH, an extraction in chloroform led to the collection of the fatty acids, which were then methylated with diazomethane and analyzed by a combined gas–liquid chromatography mass spectrometry (GLC-MS) (Santa Clara, CA, USA). The ester-bound fatty acids were released after treatment with aqueous 0.5 M NaOH, MeOH (1:1, *v*/*v*, 85 °C, 2 h), and the product was then acidified, extracted in chloroform, methylated with diazomethane, and analyzed by GLC-MS.

An aliquot of the LPS fraction (0.5 mg) was also dried and methanolized with 1 M HCl/CH_3_OH. The reaction was carried out at 80 °C for 16 h. The mixture was extracted three times with hexane. The hexane layer, containing the fatty acids as methyl esters derivatives, was analyzed by GLC-MS.

The fatty acids’ absolute configuration was established as previously reported [21]. Briefly, the 3-hydroxy fatty acids were released by strong alkaline hydrolysis in 4 M NaOH (100 °C, 5 h), converted into the 3-methoxy acid l-phenylethylamides, and then analyzed by GLC-MS. The comparison of the retention times of authentic l-phenylethylamides of various standard fatty acids with those derived from the examined LPS was crucial to assign the (R) configuration to all of the fatty acids composing the *S. salinus* M19-40^T^ LPS lipid A.

The analyses were executed on an Agilent Technologies gas chromatograph 6850A equipped with a mass selective detector 5973N and a Zebron ZB-5 capillary column (Phenomenex, 30 m × 0.25 mm internal diameter, flow rate 1 mL min^−1^, He as carrier gas). The following temperature program was employed for the lipid analysis: 140 °C for 3 min, 140 °C → 280 °C at 10 °C min^−1^.

### 4.4. Ammonium Hydroxide Hydrolysis of S. salinus M19-40^T^ Lipid A

An aliquot of lipid A (0.5 mg) was treated with 10% ammonium hydroxide, as previously reported [29]. The sample was dried and analyzed by MALDI MS.

### 4.5. MALDI MS Analysis

A reflectron MALDI-TOF MS and MS^2^ analysis was performed on an ABSCIEX TOF/TOF^TM^ 5800 Applied Biosystems mass spectrometer, equipped with an Nd:YLF laser with a λ of 345 nm, a <500 ps pulse length, and a repetition rate of up to 1000 Hz. The lipid A fraction was dissolved in chloroform/methanol (1:1, *v*/*v*), as previously described [30,31]. The matrix was the trihydroxyacetophenone (THAP) (91928-Sigma Aldrich^®^) dissolved in methanol/0.1% trifluoracetic acid/acetonitrile (7:2:1, *v*/*v*/*v*) at a concentration of 75 mg mL^−1^ [30,31]. The ammonium-treated lipid A was dissolved in chloroform-trifluoroethanol (4:1, *v*/*v*) and the matrix used was 2,5-dihydroxy benzoic acid (85707-Sigma Aldrich^®^) in acetonitrile 0.2% trifluoroacetic acid (7:3, *v*/*v*) [29]. Lipid A preparation of 0.5 μL and 0.5 μL of the matrix solution were deposited on the MALDI plate and left to dry at room temperature. Each spectrum was a result of the accumulation of 1500 laser shots, whereas 6000–7000 shots were summed for the MS^2^ data acquisitions [30,31,32]. The reported MS^2^ spectra were all acquired without collision gas. Nevertheless, MS^2^ experiments were executed with argon or air as the collision gas, and the resulting fragmentation spectra were compared with those that were obtained without collision-induced dissociation. No large differences were observed in all of the cases in the mass range above *m*/*z* 200. Below this mass, the product ions corresponding to the phosphate fragments at *m*/*z* 79 and 97 were far more abundant in the collision-induced dissociation spectra, regardless of the collision gas.

## 5. Conclusions

The structural characterization of the halophilic bacteria LPS has been—and continues to be—crucial in order to provide further insights into the molecular mechanism at the basis of the marine habitat adaptation phenomena. In this study, we have demonstrated that the moderate halophile *S. salinus* M19-40^T^ expresses a rough-type LPS and a lipid A with a peculiar chemical structure, which was characterized by a mixture of tri-, tetra-, and penta-acylated species, decorated by one or two phosphate groups. The 2 + 3 acyl chains distribution, with the respect to the glucosamine backbone, and the occurrence of the amide-bound 14:0 (3-oxo) fatty acid are uncommon structural features found for *S. salinus* M19-40^T^ lipid A. Given the rare chemical characteristics, it would be interesting to define whether they have a role in the adaptation process to the marine environment. Moreover, it would be intriguing to study the *S. salinus* M19-40^T^ lipid A immunological properties, thus furnishing important information on the LPS fine structure-immunoactivity relationship.

## Supplementary Materials

The following are available online at http://www.mdpi.com/1660-3397/16/4/124/s1. Figure S1: MALDI MS^2^ spectrum of the bis-phosphorylated tetra-acylated lipid A species at *m*/*z* 1301.8 from *S. salinus* M19-40^T^ R-LPS. Fragments assignment is reported. The proposed structure for the tetra-acylated lipid A species is reported in the inset. Figure S2: Section of the positive-ion MALDI mass spectrum of the lipid A from *S. salinus* M19-40^T^ R-LPS showing most of the lipid A species present with Na^+^ counterions. Figure S3: Negative-ion MALDI mass spectrum of the lipid A from *S. salinus* M19-40^T^ R-LPS obtained from 10% ammonium hydroxide hydrolysis. The proposed structures of the product are reported in the figure.
