# Supplementary material for: The Structure of the Lipid A from the Halophilic Bacterium Spiribacter salinus M19-40T
Source: Mar Drugs. 2018 Apr 11;16(4):124. doi: 10.3390/md16040124 (PMC5923411; doi:10.3390/md16040124)
Supplement: Supplementary file 1 [file marinedrugs-16-00124-s001.pdf]

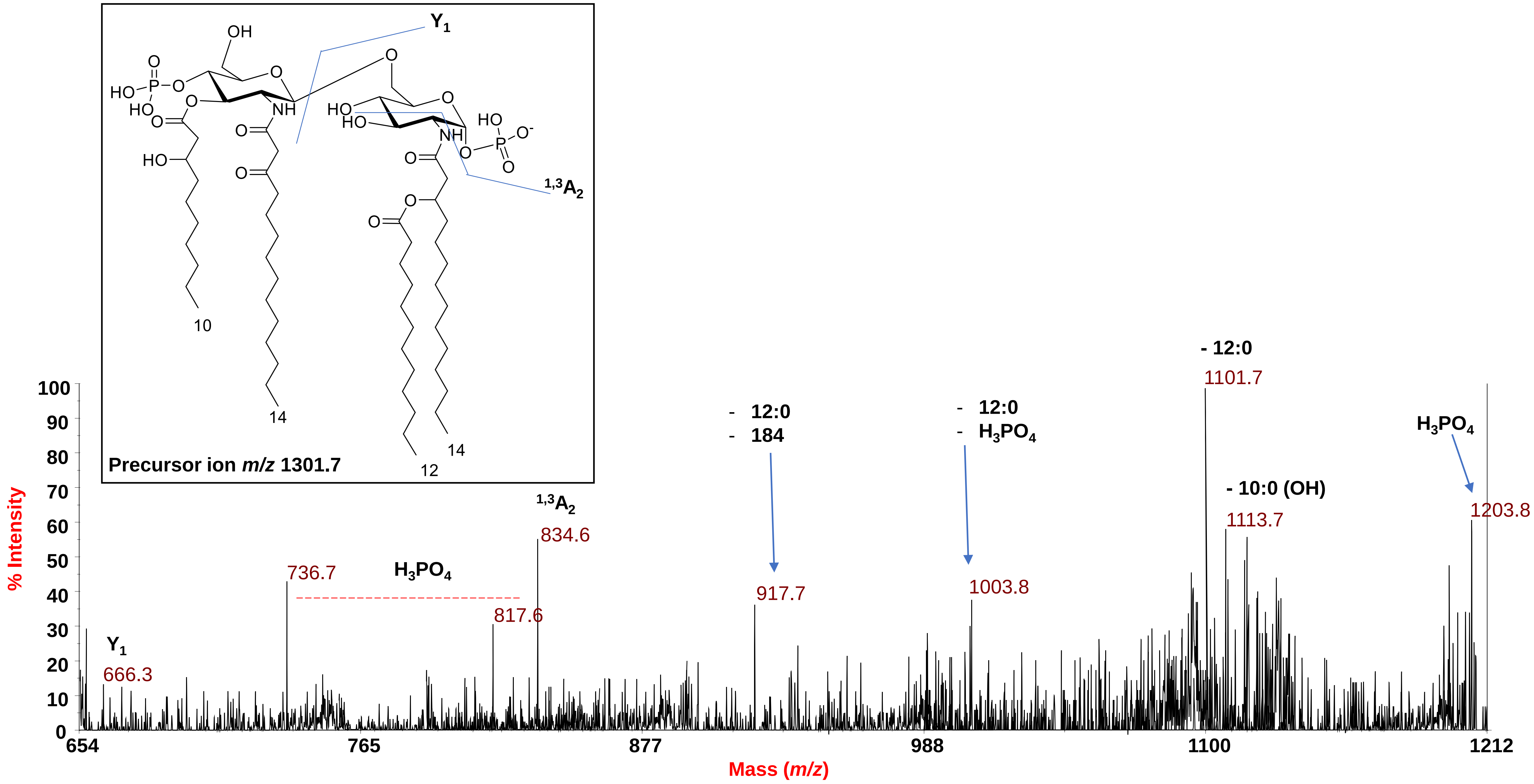

Figure S1. MALDI MS^2 spectrum of the bis-phosphorylated tetra-acylated lipid A species at *m/z* 1301.8 from *S. salinus* M19-40^T R-LPS. Fragments assignment is reported. The proposed structure for the tetra-acylated lipid A species is reported in the inset.

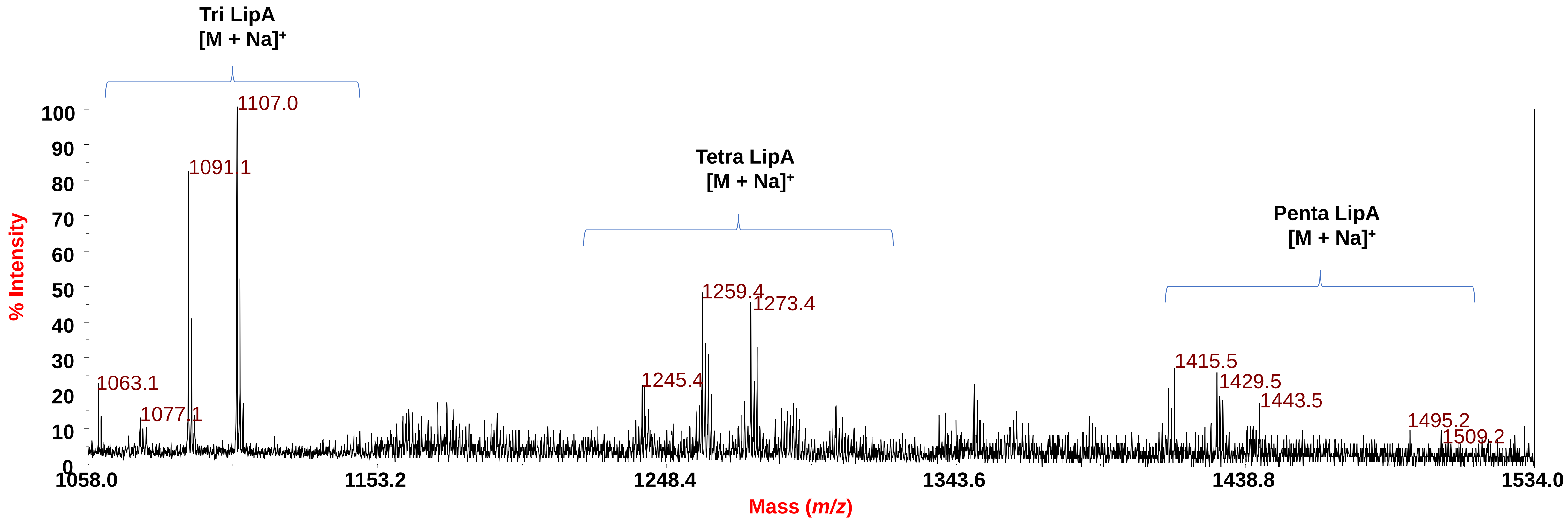

Figure S2. Section of the positive-ion MALDI mass spectrum of the lipid A from *S. salinus* M19-40^{T} R-LPS showing most of the lipid A species present with Na^{+} counterions.

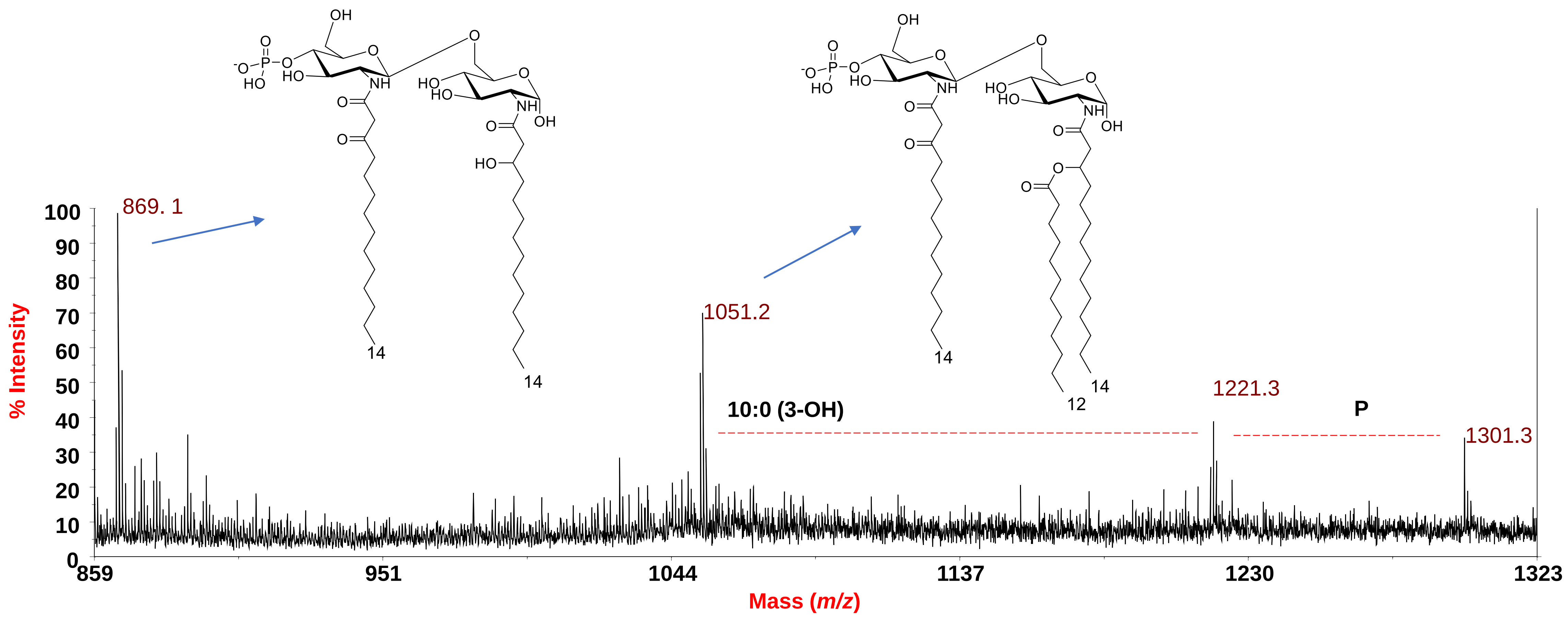

Figure S3. Negative-ion MALDI mass spectrum of the lipid A from *S. salinus* M19-40^{T} R-LPS obtained from 10 % ammonium hydroxide hydrolysis. The proposed structures of the product are reported in the figure.
